# CD8 regulatory T cell therapy in transplantation: a new path to clinical success?

**DOI:** 10.3389/ti.2026.17003

**Published:** 2026-06-09

**Authors:** Yannick D. Muller, Julien Zuber

**Affiliations:** 1 Division of Immunology and Allergy, Lausanne University Hospital and University of Lausanne, Lausanne, Switzerland; 2 Centre for Human Immunology Lausanne, Lausanne, Switzerland; 3 Département des Maladies du rein et Métabolisme, Transplantation et Immunologie Clinique, Hôpital Necker, Assistance-Publique Hôpitaux de Paris, Université Paris-Cité, Paris, France

**Keywords:** CD8 treg, cell therapy, tolerance, transplantation immunology, treg, regulatory T cell, antigen presentation

Since the first success using modified T cells redirected against B cells to treat acute lymphoblastic leukemia and the unexpected, yet durable, remission of cancer observed in Emily Whitehead, cell therapy has emerged as a novel drug masterclass [1]. In particular, the unique capabilities of T cells to actively migrate into tissues and the sites of inflammation where conventional therapies fail have opened novel strategic avenues for treating refractory and severe diseases [2]. In fact, the number of clinical trials involving cell-based therapies in medicine is in an exponential growth phase, commensurate with the expectations and anticipated therapeutic benefits associated with their outcomes [3].

Conceptually, T cells can be harnessed either to selectively deplete specific cellular subpopulations, particularly B cells, or to suppress the licensing of effector T cells. In this context, naturally occurring CD4^+^ regulatory T cells (CD4^+^ Tregs) have attracted considerable attention. To date, more than 30 clinical trials have been registered in the field of transplantation [4]. Thus, since their discovery, awarded with the 2025 Nobel Prize in Physiology or Medicine, CD4^+^ Tregs have generated substantial enthusiasm within the scientific community as they are built with dozens of suppressive mechanisms, enabling Tregs to modulate the immune response in a highly controlled and multifaceted manner [5, 6]. In transplantation, the ONE study, an investigator-led single uncontrolled arm trial performed across eight international centers, could show early safety and promising results with CD4^+^ Tregs on reducing the immunosuppressive treatment in kidney recipients [7]. The TWO study, a phase 2b, is currently ongoing to validate those results.

More recently, the Eight-Treg study is a first-in-human phase I clinical trial designed to evaluate the safety, feasibility, and early signals of efficacy of autologous CD8^+^ Treg therapy in kidney transplantation. CD8^+^ Tregs are defined by a CD8^+^CD45RC^low/−^ phenotype, a subset previously characterized by potent suppressive activity [8–11]. In this protocol, CD8^+^ Tregs are isolated from the peripheral blood of transplant candidates prior to transplantation through cell sorting, and subsequently expanded *ex vivo* under GMP conditions using anti-CD3/anti-CD28 stimulation in the presence of low-dose IL-2, IL-15, and rapamycin, conditions known to promote regulatory stability [12]. The expansion process spans 21 days, enabling the generation of clinically relevant cell numbers while preserving both the phenotypic identity and suppressive function of the cells. At the end of the culture, the cell product exhibits a stable regulatory profile, characterized by homogeneous FOXP3 expression, high GITR levels, and low CD127 expression, consistent with a *bona fide* Treg signature. The expanded cells are reinfused into the recipient the day before transplantation, *in lieu* of conventional induction therapy, and in combination with standard immunosuppressive treatment that may be adjusted according to clinical evolution. The study follows a dose-escalation design primarily aimed at assessing safety, *in vivo* persistence of the infused cells, and immunological effects, while also exploring preliminary efficacy endpoints, including graft function, incidence of rejection, and markers of immune regulation. Protocol biopsies performed at months 1 and 3 post-transplantation enable the evaluation of graft inflammation, immune cell infiltration, and the presence of regulatory signatures within the tissue. In parallel, longitudinal immunomonitoring is conducted to assess the persistence, phenotype, and functional impact of transferred CD8^+^ Tregs on the recipient immune system.

The rationale for developing CD8^+^ Treg-based therapies is supported by a growing body of experimental and translational evidence demonstrating their potent immunoregulatory capacity in transplantation and autoimmunity [13]. In both rodent and humanized models, CD8^+^ Tregs have been shown to contribute to the maintenance of immune tolerance and to prevent allograft rejection or autoimmune pathology [8, 10, 14, 15]. Mechanistically, these cells suppress pathogenic immune responses through multiple, non-redundant pathways, including IL-2 consumption, modulation of antigen-presenting cell function, and secretion of regulatory cytokines [16]. Notably, CD8^+^ Tregs recognize donor-derived antigens presented by MHC class I molecules, which are ubiquitously expressed on nucleated cells, including graft parenchymal cells [17, 18]. This feature confers upon CD8^+^ Tregs the unique ability to exert regulatory activity directly within transplanted tissues, thereby enabling local control of alloimmune responses at sites of inflammation. In addition, preclinical studies have demonstrated that CD8^+^ Tregs can be efficiently expanded *ex vivo* while maintaining stable suppressive properties [10, 12, 15], supporting their development as a clinically relevant cellular therapeutic product. Collectively, these observations provide a strong mechanistic and translational framework for the clinical evaluation of CD8^+^ Treg adoptive cell therapy as an innovative strategy to promote immune tolerance and improve long-term graft outcomes in organ transplantation.

Key differences arising from ongoing trials testing CD4^+^ and CD8^+^ Tregs may depend on the direct and indirect mechanisms of antigen presentation and alloantigen recognition pathways [19]. While some evidence suggests that the allogeneic CD8^+^ T cell repertoire is driven by immunodominant, organ-specific peptides rather than conserved regions of non-self major histocompatibility complex (MHC) molecules, the direct pathway is primarily initiated by a cellular, T-cell mediated, allorecognition [20, 21]. Accordingly, CD8^+^ Tregs, through their recognition of HLA class I molecules, are uniquely positioned to interact not only with donor and recipient antigen-presenting cells but also directly with the transplanted tissue itself (Figure 1). In contrast, CD4^+^ Tregs, which primarily target HLA class II molecules, expressed at much lower levels within the graft, are better positioned to inhibit indirect alloantigen recognition pathways. This pathway depends on the processing and presentation of allogeneic peptides by recipient dendritic cells and B cells, ultimately driving chronic antibody-mediated rejection and progressive graft dysfunction [22, 23].

**FIGURE 1:**
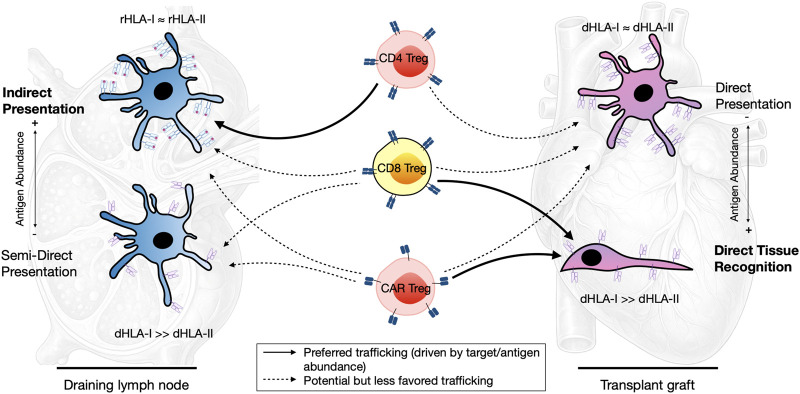
Cell-cell interactions in light of the allorecognition pathways and Treg therapy. Schematic hypothesis of preferential localization driven primarily by cognate antigen/target abundance. Abbreviations. HLA Human Leucocyte Antigen; r recipient; d donor; CAR chimeric antigen receptor.

The initiation of clinical trials evaluating CD8^+^ Tregs also echoes the recent enthusiasm surrounding the development of chimeric antigen receptor (CAR) Tregs targeting HLA-A2 molecules, which are expressed in approximately 30% of the general population [24, 25]. By exploiting HLA-A2 mismatches between donor and recipient, Tregs can be engineered to exert potent, antigen-specific suppressive activity in the presence of HLA-A2, even if the selectivity of CAR-Tregs for dendritic cells versus graft tissue or endothelial cells remains still unclear [26, 27]. Ultimately, this strategy is mainly aiming at inducing a highly suppressive local microenvironment within the graft consistent with the well-documented role of Tregs in promoting solid tumor progression and reduced immunotherapeutic efficacy in cancer [28]. It should be noted that peer-reviewed clinical outcomes from HLA-A2 CAR-Treg trials are still missing. Early safety data from the STEADFAST study, a Phase I/II clinical trial initiated by Sangamo Therapeutics, and the LIBERATE trial by Quell Therapeutics, both evaluating HLA-A2 CAR Tregs in kidney and liver transplantation respectively, have been presented at international meetings and correlated with the migration and persistence of Treg in the transplanted organs. Yet, it remains unclear whether these results will be sufficient to maintain these programs open and ultimately implemented in clinical practice considering the cost and the manufacturing complexity of those cells.

Nevertheless, the field of Treg therapy is receiving encouraging important positive signals from fields outside of SOT. The results from the Phase 3 Precision-T study indicate that donor derived Treg therapies (named Orca-T) can significantly enhances survival free from chronic graft-versus-host disease in patients with acute myeloid leukemia, acute lymphoblastic leukemia, and myelodysplastic syndromes, compared with conventional therapies following allogeneic hematopoietic stem cell transplantation. Thus, Orca-T is currently under Priority Review by the U.S. Food and Drug Administration [29]. Sonoma Biotherapeutics has announced favorable interim results from the ongoing Phase 1 REGULATE-RA study evaluating SBT-77-7101 in patients with refractory rheumatoid arthritis. The data shows encouraging safety profile alongside preliminary evidence of clinical efficacy in this treatment-resistant population.^1^

Thus, the initiation of new clinical trials in the field of SOT is not only encouraging but also critically important to maintain scientific dynamism and knowledge gain. We are convinced that such efforts will contribute to the development of more targeted immunosuppressive strategies, ultimately improving long-term patient survival and quality of life. Even if unmodified CD4^+^ or CD8^+^ Treg therapy remains insufficient to induce durable immune tolerance, the expanding repertoire of cellular engineering technologies is an impressive reservoir for future scientific innovation. Thus, Treg antigen-specificity may be easily refined by targeting both direct and indirect presentation of immunodominant allogeneic peptides, including through TCR engineering [30], CD4-to-CD8 co-receptor swapping strategies [31], or by the development of antigen/tissue-specific CARs with optimized signaling domains [32, 33]. The incorporation of supraphysiological functional properties, such as controlled release of Treg pro-survival cytokines [34], is another example that could substantially augment Treg persistence and therapeutic efficacy in the coming years.

## Data Availability

The original contributions presented in the study are included in the article/supplementary material, further inquiries can be directed to the corresponding author.

## Footnotes

1
https://acrabstracts.org/abstract/a-phase-1-study-of-autologous-car-treg-cells-in-refractory-rheumatoid-arthritis-interim-report-of-safety-and-efficacy/
